# Determinants of comprehensive knowledge on mother-to-child transmission of HIV and its prevention among childbearing women in Rwanda: insights from the 2020 Rwandan Demographic and Health Survey

**DOI:** 10.1186/s12889-022-14925-9

**Published:** 2023-01-03

**Authors:** Michael Deynu, Jerry John Nutor

**Affiliations:** 1 Ho Municipal Hospital, Ho, Volta Region Ghana; 2 Family Health Care Nursing Department, School of Nursing, University of California, San Francisco, 2 Koret Way, CA San Francisco, USA

**Keywords:** Reproductive age, Sub-sharan Africa, Vertical transmission, Counseling, Antiretroviral treatment, Mother-to-child transmission, HIV

## Abstract

**Background:**

Maternal knowledge on mother-to-child transmission (MTCT) and its prevention has been identified to enhance maternal testing and adherence to antiretroviral therapy (ART) regimen. Examining prevalence and associated factors on MTCT and its prevention among women provides empirical evidence for design and implementation of health strategies aimed at increasing MTCT knowledge and its elimination. This study therefore examined women’s comprehensive knowledge and associated factors on MTCT and its prevention among childbearing women in Rwanda.

**Methods:**

Analysis was conducted on a weighted sample of 14,634 women from the 2020 Rwanda Demographic and Health Survey (RDHS). Dataset cleaning and missing value analysis was conducted. Chi square, bivariate and multivariable regression was then conducted in complex samples in SPSS. Alpha level set at *p* < 0.05 and at 95% Confidence Interval (95% CI). All analysis were adjusted for unequal probability sampling using survey weights. Bivariate and multivariable results were reported with crude and adjusted odds ratios.

**Results:**

The mean age was 29.2 years, SD-9.1. Prevalence of HIV testing and comprehensive knowledge on MTCT and its prevention among women in Rwanda was 79.6% and 65.1% respectively. Findings from this study showed that married women have higher odds (aOR = 1.18, 95% CI = 1.04–1.35) of comprehensive knowledge on MTCT and its prevention compared to those unmarried. Women who were living in southern (aOR = 1.23, 95%CI = 1.02–1.48) and eastern (aOR = 1.37, 95% CI = 1.13–1.66) parts of Rwanda were more likely to have adequate knowledge on MTCT of HIV and its prevention than those in Kigali. Also, women who received post-test counselling (aOR = 1.26, 95% CI = 1.01–2.11) have increased knowledge on MTCT than those who did not. Women with access to radio (aOR = 1.18, 95% CI = 1.06–1.32) and television (aOR = 1.25, 95% CI = 1.07–1.45) at least once a week were more likely to have adequate knowledge on MTCT and its prevention compared to those who do not in Rwanda.

**Conclusion:**

There is inadequate knowledge on MTCT and its elimination among women of reproductive age in Rwanda. Strategies to enhance knowledge on MTCT and its prevention among childbearing women should be adopted through rigorous educational sensitization campaigns using local media such as radio and television. Health services that focus on prevention of MTCT must emphasize post-test counselling.

## Background

Globally, Human Immunodeficiency Virus (HIV) infection continues to pose significant public health burden to nations and territories most especially in Lower-and-Middle-Income Countries (LMICs) [1] despite the introduction of antiretroviral (ARVs), and elimination of mother-to-child transmission of HIV (EMTCT) services [2]. The global burden of HIV was 38.4 million with over 1.5 million new infections, and with 650,000 deaths in 2021 [3]. Sub-Saharan Africa (SSA) remains the most devastatingly affected region with about 67% of the global burden of HIV and women in their reproductive years are most affected with more than half of the people living with HIV being women [4].

In 2020, Rwanda, a SSA country, recorded HIV prevalence of 3.0% and 2.6% among adults aged 15–64 years and 15–49 years respectively, [5–7]. Mother-to-child transmission (MTCT) which is also referred to as vertical transmission of HIV has been identified as the main source of HIV infection amongst children under age 15 years. The global burden of pediatric HIV infection stood at 1.7 million children under 15 years with associated 98 000 mortalities in 2021 [3]. MTCT occurs when an HIV positive mother passes the virus to the child during pregnancy, labor, delivery or through breastfeeding [8, 9], and in Rwanda the national prevalence of MTCT of HIV stood at 1.56% in 2019 [7]. In sub-Saharan Africa, an estimated 180,000 new HIV infections occurred in 2017 among children aged 0–14 years predominantly during breastfeeding. Again, it’s been reported that risk of HIV transmission between mother and the child ranges 15-45% (that is, 5–10% during pregnancy, 10–20% during labor and childbirth and 10–20% through mixed infant feeding) in the absence of any intervention to eliminate MTCT, [10, 11]. Infants and young children infected with HIV have a substantial mortality and morbidity risks which is increased especially during pregnancy and labor with nearly half of these children dying before their second birthday without any treatment and intervention [3].

The World Health Organization (WHO) recommendations to reducing MTCT includes; prevention of HIV among women of reproductive age, prevention of unintended pregnancies among women living with HIV, and provision of antiretroviral therapy (ARTs) to mothers living with HIV [1, 11]. In addition, the Joint United Nations Programme on HIV/AIDS (UNAIDS) launched the 95-95-95 strategy in 2014 with its focus to ensuring that 95% of all persons living with HIV knows their status, 95% of those diagnosed as positive cases receive ARTs and 95% of those on ARTs achieve viral suppression by 2030 [12]. Remarkable efforts have been made by countries towards the elimination of MTCT of HIV through implementation and utilization of ART, and prevention of MTCT of HIV programs [4]. Rwanda has also made enviable progress in increasing uptake of EMTCT services by pregnant women through initiation of ARTs regardless of their CD4 cell counts, ensuring that health facilities were offering EMTCT services, pregnant women attended a minimum of one Antenatal Care (ANC) visit, and lastly, pregnant women who were infected received ARTs for EMTCT of HIV [13]. Despite these interventions, the rate of MTCT of HIV among women in Rwanda at stood at 1.5% [7]. This may be due to interplay of several factors including limited resources in designing and implementing EMTCT services, both human and capital logistics and stigma associated with its transmission.

Previous studies in Zimbabwe [2], Tanzania [8] and Ethiopia [14, 15] have reported the importance of women’s knowledge on EMTCT of HIV in eliminating pediatric HIV infection. Again, comprehensive maternal knowledge on EMTCT of HIV has been found to be associated positively with uptake of maternal HIV testing [8], and ART adherence [9, 11]. This is because childbearing women with adequate knowledge on HIV and EMTCT tend to protect themselves and their families from HIV infection and are highly likely to seek testing and treatment compared to those who have less knowledge on MTCT [16]. Many theories including health belief model, theory of reasoned action amongst others have provided framework for HIV studies in recent past [17]. The Social Cognitive Theory [18] and Information Motivation Behavioral Skills (IMB) [19] was however adapted in this current study because they provided holistic framework to understanding behavioral change, emphasizing that knowledge on a disease (what the disease is, its transmission and prevention) and health information, motivation and behavioral skills are essential to understanding and enhancing behavioral change, in addition to intentions to adopt preventive measures and achieve desired outcomes of protection from the disease or illness [20].

In Rwanda, some studies have been conducted around HIV and its prevalence amongst others [5, 13] [21–24]. The focus of these studies has largely been identifying HIV incidence and prevalence at the district level [5], risk factors and characteristics of mothers living with HIV [13] and rates of MTCT of HIV at 6–10 weeks postpartum [21] amongst others. The findings of these studies remain relevant to population health and development. Nonetheless, to the best of knowledge of the authors, no study was conducted on comprehensive maternal knowledge on MTCT and PMTCT of HIV among reproductive women using a large population survey that is nationally representative in Rwanda. Therefore, within the Rwandan context, very little is known about the prevalence and influencing factors of comprehensive maternal knowledge on MTCT and PMTCT of HIV among this age cohort using large population and nationally representative data. In view of this, the current study aimed to examine associated determinants of comprehensive knowledge on MTCT and PMTCT of HIV among women in their reproductive age in Rwanda.

## Methods

### Study design and data source

This is secondary data analysis of a cross sectional study carried out on women of reproductive age 15–49 years in Rwanda. The data was extracted from the 2020 Rwandan Demographic and Health Surveys (RDHS) conducted from November 2019-July 2020. The 2020 RDHS was a follow up to the previous 2014/2015 RDHS [25]. Its primary objective was to provide up-to-date estimates of basic demographic and health indicators including information on fertility levels and preferences, contraceptive use, maternal and child health, infant, child and neonatal mortality levels, maternal mortality, gender, nutrition, awareness about HIV/AIDS, self-reported Sexually Transmitted Infections (STIs) and other health indicators that are relevant to the attainment of the Sustainable Development Goals (SDGs) [25]. The survey respondents in the 2020 RDHS were representatively selected from the five (5) regions of Rwanda. The 2020 RDHS followed a two-stage sample design that allows estimates of key indicators at the national level as well as for urban and rural areas, five regions or provinces. The first stage involved the selection of sample points (clusters) consisting of Enumeration Areas (EAs). A total of 500 clusters were selected, 112 in urban and 388 in rural areas.

The second stage involved systematic sampling of households within the selected clusters providing a sampling frame from where the households were randomly selected from all the clusters to provide estimates for key indicators. A total sample size of 14,634 women aged 15–49 years were interviewed in the 2020 RDHS. Therefore, sample size analyzed in this study was 14,634 respondents. The detailed description of the methodology of the survey design, survey tools used, methodology and the data collection methods for the 2020 RDHS has been described in the RDHS report [26].

### Study setting

This study was conducted in Republic of Rwanda, a landlocked country located in the Great Rift Valley of Eastern-Central Africa. It has a total area of 26 338 sq. km and bounded to the north by Uganda, to the east by Tanzania, and the west by Democratic Republic of Congo with an estimated population of 13 256 000 in 2022. It has five provinces namely North, East, West, South and Kigali (Capital).

### Study variables and measurements

#### Outcome variable

The main outcome variable in this study is reproductive women’s comprehensive knowledge on MTCT and EMTCT of HIV in Rwanda. It is a composite score of five [5] different questions that were similar to those in previous studies [2, 4, 8, 10, 14]. The questions included (i) “Now I would like to talk about something else, have you ever heard of HIV/AIDS?”, (ii) “Can HIV be transmitted from mother to her baby during pregnancy?”, (iii) “Can HIV be transmitted from the mother to her baby during delivery?”, (iv) “Can HIV be transmitted from the mother to her baby during breastfeeding?”, (v) “Are there any special drug or medicines that a doctor or a nurse can give to a woman infected with HIV to reduce the risk of transmission to the baby?”. Responses to each of these questions were coded as 1 if the respondent answered “yes” and 0 if the respondent answered “no”. An aggregate score was then computed and a score of (5) meant the respondent had adequate knowledge on MTCT and EMTCT of HIV whilst a score less than (5) by a respondent was considered as having inadequate knowledge on MTCT and EMTCT of HIV. A binary variable was therefore created based on the aggregate scores.

### Independent variables

The Social Cognitive Theory [18] and the Information Motivation Behavioral Skills Theory (IMB) [19] and previous literature [1, 2, 4, 8, 10, 11] provided the theoretical foundation and guided selection of the independent predictors analyzed in this study respectively. The independent predictors have been categorized into Individual level factors such as Respondents age (15–19, 20–24, 25–29, 30–34, 35–39, 40–44, 45–49), Marital status (recoded into Never in union/Not Married, Married/Living with partner, Divorced/Separated/Widowed), Highest educational level (No education, Primary, Secondary, Higher), Religion (Catholics, Protestants, Adventists, Muslim, Traditional & Others), Frequency of reading newspaper/magazine (Not at all, Less than once a week, At least once a week), Frequency of listening to radio (Not at all, Less than once a week, At least once a week), Frequency of watching Television (Not at all, Less than once a week, At least once a week), Wealth Index Combined (Poorest, Poorer, Middle, Richer, Richest), Currently pregnant (No or Unsure, Yes), Visited health facility last 12 months (No, Yes), Covered by Health Insurance (No, Yes), Respondent currently working/Employment status (Employed, Unemployed). The Community level variables included Region of residence (Kigali, South, West, North, and East) and Type of Place of residence (Urban, Rural). Others included HIV related knowledge variables; Ever been tested for HIV in last 12 months prior to the survey (No, Yes), Knows a place to get HIV test (No, Yes), Knowledge and use of HIV self-test kits (Never heard of HIV test kits, Knows and self-tested), Tested for HIV as part of ANC visit (No, Yes), Got results for HIV as part of ANC visit (No, Yes), Received counselling after being tested for HIV as part of ANC visit (No, Yes), Received results for last HIV test (No, Yes).

### Data analysis

The data in the current study was analyzed through dataset cleaning and integrity checks to ensure completeness and consistency of the dataset. Univariate analysis as well as missing data analysis was conducted. Bivariate and multivariable regression analysis was then conducted using IBM Statistical Software for Social Sciences (SPSS) version 26, level of significance set at *p* < 0.05 and at 95% Confidence Interval (95%CI). Survey characteristics were described using frequencies and percentages. Pearson’s Chi square was then performed to investigate the associations between the dependent variable and the predictors of MTCT and EMTCT of HIV. The multivariable logistic regression model was then fitted with the independent variables to identify the determining factors of comprehensive MTCT and EMTCT of HIV knowledge. This was to adjust for confounders [27, 28].

The multivariable analysis was conducted through a three-stage modelling to understand the factors that influence comprehensive MTCT and EMTCT of HIV Knowledge. The first model involved the sociodemographic or individual level characteristics of the survey respondents. The second modeling entailed both individual level factors and community level factors. The third modelling involved the second model and HIV related knowledge factors to produce adjusted odds ratios that are independently associated with reproductive women’s knowledge on MTCT and EMTCT of HIV. The bivariate analysis results were reported in crude odds ratios (COR) whilst the multivariable results were reported with adjusted odds ratios (aOR) at 95% Confident Intervals (95%CI). Sample weights were applied to account for sampling biases. All analysis were conducted through the complex samples analysis in SPSS after the Complex Samples Analysis Plan (CS Plan) was generated in SPSS using the weight, cluster and strata variables in the Rwanda DHS. This allowed for the adjustment of weight, stratification and clustering of the sampling design in order to produce national estimates that are representative of the general population taking into account the weights for under or over sampling of specific groups in Rwanda [26, 29–31].

### Ethical consideration

The study was performed in accordance with the Declaration of Helsinki and approved by appropriate ethics committee. Ethical clearance was obtained from the Rwanda National Ethics Committee and the ICF Institutional Review Board. Informed consents were obtained from participants prior to data collection. We obtained permission from the DHS program to use the 2020 RDHS for our study at https://dhsprogram.com/data/available-datasets.cfm. All data were anonymized before the authors received the data. All methods were performed in accordance with the relevant guidelines and regulations.

## Results

### Sociodemographic characteristics of survey participants

In Table 1 below, a total sample of 14,634 reproductive women from the RDHS dataset was analyzed in this study. The mean age was (M = 29.2years, SD = 9.1). Almost a quarter (22.3%), of the respondent were aged 15–19 years while only 8.3% were 45–49 years old. Majority, (80%) of the participants were residing in rural areas whilst about 19% were found in the urban areas. About 27% of them were from the eastern part of Rwanda whilst Kigali which is the capital city recorded the least (14.8%) of survey participants (Table 1). More than half (58.3%) had at least primary education whereas those with secondary and higher education accounted for 27.9% and 4.4% respectively. Approximately, one out of ten participants (9.4%) had no education. Furthermore, over 20% of them were found in the richer and richest quantiles whilst over 18% were poorest. Majority of the participants (94.1%) were not pregnant.

**Table 1:** Sociodemographic characteristics of reproductive women (15–49 years) in Rwanda, *N* = (14,634)

Characteristics of the respondents	N (%)	Comprehensive MTCT and EMTCT of HIV Knowledge		
		Inadequate MTCT and EMTCT knowledge on N (%)	Adequate MTCT and EMTCT Knowledge N (%)	*P*-values
**Age** (M = 29.2, SD = 9.1)				< 0.001
15–19	3258(22.3)	1265(24.8)	1993(20.9)	
20–24	2414(16.4)	862(16.9)	1552(16.3)	
25–29	2073(14.1)	700(13.7)	1373(14.4)	
30–34	2118(14.5)	689(13.5)	1429(15.0)	
35–39	2072(14.2)	657(12.9)	1415(14.8)	
40–44	1488(10.2)	506(9.9)	982(10.3)	
45–49	1211(8.3)	422(8.3)	789(8.3)	
**Current marital status**				< 0.001
Never in union/Not married	5914(40.4)	2295(45.0)	3620(38.0)	
Married/Living with partner	7401(50.6)	2390(46.9)	5011(52.6)	
Divorced/Separated/Widowed	1319(9.0)	416(8.1)	902(9.4)	
**Highest educational level attained**				< 0.001
No education	1377(9.4)	437(8.6)	939(9.9)	
Primary	8529(58.3)	2908(57.0)	5621(59.0)	
Secondary	4086(27.9)	1471(28.8)	2616(27.4)	
Higher	642(4.4)	285(5.6)	357(3.7)	
**Religion**				0.08
Catholics	5363(36.7)	1813(35.6)	3551(37.2)	
Protestants	6905(47.2)	2492(48.8)	4413(46.3)	
Adventists	1836(12.5)	608(11.9)	1227(12.9)	
Muslim	269(1.8)	90(1.8)	179(1.9)	
Traditional&Others	261(1.8)	98(1.9)	163(1.7)	
**Wealth Index**				0.05
Poorest	2741(18.7)	921(18.1)	1819(19.1)	
Poorer	2756(18.8)	934(18.3)	1822(19.1)	
Middle	2757(18.9)	950(18.6)	1807(19.0)	
Richer	2966(20.3)	1020(20.0)	1947(20.4)	
Richest	3414(23.3)	1276(25.0)	2138(22.4)	
**Region of residence**				< 0.001
Kigali	2166(14.8)	821(16.1)	1345(14.1)	
South	3065(20.9)	1026(20.1)	2039(21.4)	
West	3174(21.7)	1211(23.8)	1963(20.6)	
North	2226(15.2)	786(15.4)	1440(15.1)	
East	4003(27.4)	1257(24.6)	2746(28.8)	
**Type of place of residence**				0.41
Urban	2909(19.9)	1040(20.4)	1869(19.6)	
Rural	11,725(80.1)	4061(79.6)	7664(80.4)	
**Employment status**				< 0.001
Not Employed	4932(33.7)	1820(35.7)	3112(32.6)	
Employed	9702(66.3)	3280(64.3)	6422(67.4)	
**Covered by health insurance**				0.20
No	2539(17.4)	853(16.7)	1686(17.7)	
Yes	12,095(82.6)	4248(83.3)	7847(82.3)	
**Currently pregnant**				0.24
No or unsure	13,764(94.1)	4816(94.4)	8949(93.9)	
Yes	870(5.9)	285(5.6)	584(6.1)	
**Visited health facility last 12 months**				0.01
No	5646(38.6)	2045(40.1)	3601(37.8)	
Yes	8988(61.4)	3056(59.9)	5932(62.2)	
**Frequency of reading Newspaper**				0.62
Not at all	10,788(73.7)	3764(73.8)	7024(73.7)	
Less than once a week	2582(17.6)	882(17.3)	1700(17.8)	
At least once a week	1264(8.7)	455(8.9)	808(8.5)	
**Frequency of watching Television**				0.09
Not at all	8111(55.4)	2894(56.7)	5216(54.7)	
Less than once a week	3655(25.0)	1208(23.7)	2448(25.7)	
At least once a week	2868(19.6)	999(19.6)	1869(19.6)	
**Frequency of listening to Radio**				< 0.001
Not at all	2916(19.9)	1099(21.5)	1818(19.1)	
Less than once a week	2613(17.9)	886(17.4)	1726(18.1)	
At least once a week	9105(62.2)	3115(61.1)	5990(62.8)	
**Ever tested for HIV in last 12 months**				< 0.001
No	2989(20.4)	1209(23.7)	1779(18.7)	
Yes	11,645(79.6)	3891(76.3)	7755(81.3)	
**Knows a place to test for HIV**				< 0.001
No	406(2.8)	204(4.0)	203(2.1)	
Yes	14,228(97.2)	4897(96.0)	9330(97.9)	
**Knowledge and use of HIV self-test kits**				0.08
Never heard of HIV self-test kits				
Heard and knows how to use HIV self-test kits	12,060(82.4)	4249(83.3)	7811(81.9)	
**Tested for HIV as part of ANC visit**	2574(17.6)	852(16.7)	1722(18.1)	< 0.001
No	10,000(68.3)	3680(72.1)	6320(66.3)	
Yes	4634(31.7)	1421(27.9)	3213(33.7)	
**Got results for HIV test as part of ANC visit**				< 0.001
No	10,019(68.5)	3686(72.3)	6333(66.4)	
Yes	4615(31.5)	1414(27.7)	3201(33.6)	
**Received counselling after being tested for HIV during ANC visit**				< 0.001
No	10,472(71.6)	3851(75.5)	6622(69.5)	
Yes	4162(28.4)	1250(24.5)	2911(30.5)	
**Received results for last HIV test taken**				< 0.001
No	3217(22.0)	1304(25.6)	1913(20.1)	
Yes	11,417(78.0)	3797(74.4)	7620(79.9)	

*CI* Confidence Interval

*p* < 0.05, *OR* Odds Ratio

**Fig. 1:**
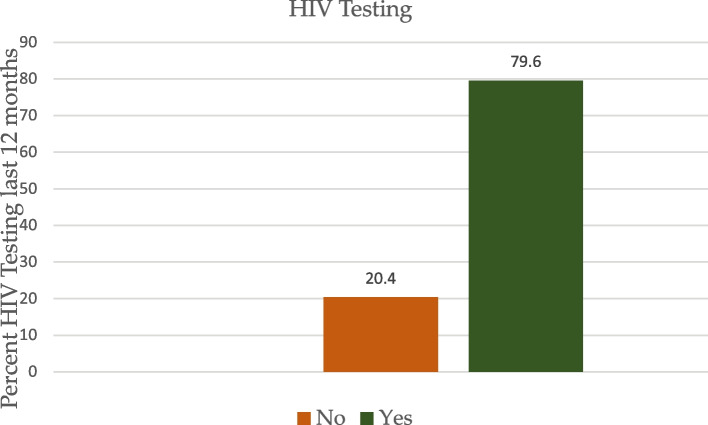
Percentage of reproductive women aged 15–49 years who have tested for HIV in the last 12 months prior to the survey

In addition, majority of the study population were Protestants and Catholics representing 47.2% and 36.7% respectively. A little over half (50.6%) of the participants were married and 66.3% were employed. Majority (82.6%) of the women were insured and over 61% had visited the health facility within the last 12 months. Almost two-third (73.7%) of the participants have not read any newspaper or magazine whereas about 62% have listened to radio and 55.4% have not watched television (TV) at all. A majority (79.6%), of the survey participants been tested for HIV in the last 12 months prior to the survey with 78.0% of them receiving results for their test (Fig. 1). Again, 82.4% of the study sample have no knowledge on HIV self-testing whilst 17.6% have some knowledge and knows how to self-test.

Furthermore, one-fifth (20.9%) of the respondents with adequate knowledge on MTCT were aged 15–19 years. More than half (52%) of the women with comprehensive knowledge were married with nearly 80% residing in rural areas. Also 64% of the participants have comprehensive MTCT and PMTCT knowledge and were employed. Again, 81.3% of the survey respondents who have knowledge on MTCT have tested within the last 12 months and 97.9% knew where to procure HIV test. More so, 83.3% of them have limited MTCT knowledge and were unaware of how to use HIV self-test kits.

### Reproductive women’s comprehensive knowledge on MTCT and EMTCT of HIV

**Fig. 2:**
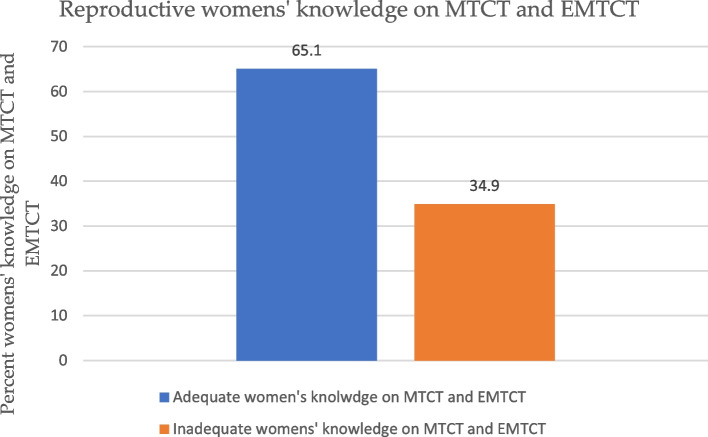
Percentages of comprehensive knowledge on MTCT and EMTCT of HIV among women aged 15–49 years in Rwanda

Table 2 below provides summary of the proportions of reproductive women’s comprehensive knowledge on MTCT and EMTCT of HIV in Rwanda. Almost all the respondents 99.7% have heard of HIV/AIDS. About 72.7% of the women in the study were aware that HIV can be transmitted during pregnancy, 94.4% believed that HIV can be transmitted during delivery. Again, of the sample population, 92.1% of the women were aware that HIV could be transmitted during breastfeeding whilst 93.5% thought that there were drugs that can be administered to avoid the transmission of HIV to the baby during pregnancy. In addition, 65.1% of the women demonstrated comprehensive knowledge on MTCT and EMTCT of HIV whilst 34.9% indicated inadequate knowledge (Fig. 2).

**Table 2:** Reproductive women’s comprehensive knowledge on MTCT and EMTCT of HIV among women aged 15–49 years in Rwanda, *N* = (14,634)

**Knowledge on MTCT and EMTCT of HIV**	**Number of respondents**	**Percentages**
**Ever heard of HIV/AIDS**		
No	41	0.3
Yes	14,593	99.7
**HIV is transmitted during Pregnancy**		
No	3998	27.3
Yes	10,636	72.7
**HIV is transmitted during delivery**		
No	823	5.6
Yes	13,811	94.4
**HIV is transmitted by breastfeeding**		
No	1150	7.9
Yes	13,484	92.1
**Drugs to avoid HIV transmission to the baby during pregnancy**		
No	946	6.5
Yes	13,688	93.5
**Comprehensive knowledge on MTCT and EMTCT of HIV**		
No	5101	34.9
Yes	9533	65.1

### Determinants of childbearing women’s knowledge on MTCT and EMTCT of HIV

Table 3 below illustrates the determining factors of the women’s comprehensive knowledge on MTCT and EMTCT of HIV in Rwanda. Findings from the bivariate analysis showed that almost all the variables included in the analysis were associated with MTCT and EMTCT of HIV knowledge. However, after controlling for confounding in the multivariate regression analysis, marital status (*p* = 0.01), region of residence (*p* = 0.00), frequency of watching TV (*p* = 0.01), frequency of listening to radio (*p* = 0.00), knowledge and use of HIV self-test kits (*p* = 0.02) and receiving counselling after being tested for HIV during ANC visit (*p* = 0.04) were found to be independently associated with MTCT and EMTCT knowledge. Married women and those who had divorced or separated were identified to have adequate knowledge (aOR = 1.18, 95%CI = 1.04–1.35) and (aOR = 1.26, 95%CI = 1.06–1.49) respectively on MTCT and its prevention compared to those unmarried.

**Table 3:** Associated factors of comprehensive knowledge on MTCT and EMTCT of HIV among reproductive women aged 15–49 years in Rwanda, *N* = 14,634

Characteristics	Crude ORs	95%CI	*P*-values	Adjusted ORs	95%CI	*P*-values
**Age**			0.00			0.79
15–19	[1,1	[1,1]		[1,1]	[1,1]	
20–24	1.14	1.01–1.28		0.95	0.83–1.09	
25–29	1.24	1.09–1.41		0.94	0.79–1.10	
30–34	1.31	1.16–1.49		0.95	0.80–1.14	
35–39	1.36	1.21–1.54		0.97	0.81–1.15	
40–44	1.23	1.08–1.40		0.89	0.73–1.07	
45–49	1.18	1.02–1.37		0.87	0.71–1.07	
**Current marital status**			0.00			0.01
Never in union/Not married	[1,1]	[1,1]		[1,1]	[1,1]	
Married/Living with partner	1.31	1.23–1.43		1.18	1.04–1.35	
Divorced/Separated/Widowed	1.37	1.19–1.58		1.26	1.06–1.49	
**Highest educational level attained**			0.00			0.00
No education	[1,1]	[1,1]		[1,1]	[1,1]	
Primary	0.90	0.79–1.02		0.90	0.78–1.04	
Secondary	0.82	0.71–0.96		0.84	0.70–1.02	
Higher	0.58	0.46–0.73		0.52	0.40–0.68	
**Religion of respondent**			0.93			0.04
Catholics	[1,1]	[1,1]		[1,1]	[1,1]	
Protestants	0.90	0.82–0.98		0.88	0.81–0.96	
Adventists	1.03	0.91–1.16		1.00	0.88–1.13	
Muslim	1.02	0.77–1.33		0.96	0.73–1.26	
Traditional&Others	0.84	0.61–1.16		0.83	0.61–1.14	
**Wealth Index combined**			0.10			0.05
Poorest	[1,1]	[1,1]		[1,1]	[1,1]	
Poorer	0.98	0.87–1.11		0.98	0.86–1.12	
Middle	0.96	0.84–1.09		0.92	0.80–1.05	
Richer	0.96	0.85–1.09		0.89	0.77–1.02	
Richest	0.84	0.74–0.96		0.77	0.64–0.93	
**Region of residence**			0.00			0.001
Kigali	[1,1]	[1,1]		[1,1]	[1,1]	
South	1.21	1.02–1.43		1.23	1.02–1.48	
West	0.98	0.83–1.16		1.02	0.84–1.22	
North	1.18	0.94–1.32		1.16	0.95–1.42	
East	1.33	1.12–1.58		1.37	1.13–1.66	
**Type of place of residence**			0.41			0.03
Urban	[1,1]	[1,1]		[1,1]	[1,1]	
Rural	1.05	0.93–1.17		0.85	0.74–0.98	
**Employment status**			0.00			0.53
Not Employed	[1,1]	[1,1]		[1,1]	[1,1]	
Employed	1.14	1.06–1.23		1.02	0.94–1.12	
**Covered by health insurance**			0.20			0.51
No	[1,1]	[1,1]		[1,1]	[1,1]	
Yes	0.93	0.84–1.03		0.96	0.86–1.07	
**Currently pregnant**			0.24			0.78
No or unsure	[1,1]	[1,1,]		[1,1]	[1,1]	
Yes	1.10	0.93–1.29		1.02	0.86–1.22	
**Visited health facility last 12 months**			0.01			0.92
No	[1,1]	[1,1]		[1,1]	[1,1]	
Yes	1.10	1.02–1.19		0.99	0.91–1.08	
**Frequency of reading newspaper**			0.57			0.59
Not at all	[1,1]	[1,1]		[1,1]	[1,1]	
Less than once a week	1.03	0.93–1.14		1.05	0.93–1.18	
At least once a week	0.95	0.80–1.11		1.06	0.89–1.26	
**Frequency of watching television**			0.07			0.01
Not at all	[1,1]	[1,1]		[1,1]	[1,1]	
Less than once a week	1.12	1.01–1.24		1.09	0.98–1.22	
At least once a week	1.03	0.92–1.16		1.25	1.07–1.45	
**Frequency of listening to radio**			0.00			0.00
Not at all	[1,1]	[1,1]		[1,1]	[1,1]	
Less than once a week	1.17	1.04–1.33		1.14	1.00–1.29	
At least once a week	1.16	1.05–1.28		1.18	1.06–1.32	
**Ever tested for HIV in last 12 months**			0.00			0.24
No	[1,1]	[1,1]		[1,1]	[1,1]	
Yes	1.35	1.23–1.48		0.82	0.60–1.13	
**Knows a place to test for HIV**			0.00			0.06
No	[1,1]	[1,1]		[1,1]	[1,1]	
Yes	1.53	1.23–1.90		1.25	0.98–1.59	
**Knowledge and use of HIV self-test kits**			0.08			0.02
Never heard of HIV self-test kits	[1,1]	[1,1]		[1,1]	[1,1]	
Heard and knows how to use HIV self-test kits	1.10	0.98–1.22		1.13	1.01–1.26	
**Tested for HIV as part of ANC visit**			0.00			0.63
No	[1,1]	[1,1]		[1,1]	[1,1]	
Yes	1.31	1.21–1.42		1.28	0.45–3.60	
**Got results for HIV test as part of ANC** visit			0.00			0.57
No	[1,1]	[1,1]		[1,1]	[1,1]	
Yes	1.31	1.21–1.42		0.74	0.26–2.11	
**Received counselling after being tested for HIV during ANC visit**			0.00			0.04
No	[1,1]	[1,1]		[1,1]	[1,1]	
Yes	1.35	1.24–1.47		1.26	1.01–1.58	
**Received results for last HIV test taken**			0.00			0.06
No	[1,1]	[1,1]		[1,1]	[1,1]	
Yes	1.36	1.24–1.50		1.34	0.98–1.83	

*CI* Confidence Interval

*p* < 0.05, OR = Odds Ratio

Similarly, women residing in Southern (aOR = 1.23, 95% CI = 1.02–1.48) and Eastern (aOR = 1.37, 95% CI = 1.13–1.66) regions of Rwanda demonstrated increased and adequate knowledge on MTCT and EMTCT. Women who have ever heard of HIV test kits and knows how to use them were found to have adequate knowledge (aOR = 1.13, 95% CI = 1.01–1.26) on MTCT and EMTCT of HIV. This study also found a positive association between post-test counselling and women’s MTCT knowledge. Women who had received counselling (aOR = 1.26, 95% CI = 1.01–2.11) have increased knowledge on EMTCT than those who do not. In addition, access to media have been found to be positively associated with maternal knowledge on MTCT and EMTCT of HIV in Rwanda. Women who have watched TV at least once a week (aOR = 1.25, 95%CI = 1.07–1.45) and listened to radio at least once a week (aOR = 1.18, 95% CI = 1.06–1.32) have demonstrated compressive knowledge compared to those who do not.

Furthermore, women in rural areas were found to be less likely (aOR = 0.85, 95% CI = 0.74–0.98) to have comprehensive maternal knowledge on mother-to-child transmission of HIV as well as those from the richest households (aOR = 0.77, 95% CI = 0.64–0.93) compared to those in the urban areas and poorest quantiles respectively in Rwanda.

## Discussion

One major hindrance to the attainment of EMTCT is the inadequate women’s knowledge on MTCT and its elimination. This study therefore examined prevalence and associated factors of reproductive women’s knowledge on MTCT and EMTCT of HIV in Rwanda. The prevalence of women’s knowledge on MTCT of HIV in this study is consistent with prevalence reported in studies in Zimbabwe [2] and northern Ethiopia [16]. However, the prevalence in our study was higher than reported in Ethiopia [14], in Mecha district in Ethiopia [32] and in Tanzania [8] however, lower than the estimate reported in North West Ethiopia [11]. This may be due to the fact that some of these studies were conducted at institutional levels where access to health education, information and knowledge on MTCT is likely higher. Majority of women in Rwanda have adequate knowledge on HIV/AIDS. Knowledge on MTCT of HIV is almost universal where women were aware HIV can be transmitted through pregnancy, delivery, and breastfeeding.

The variations identified in the prevalence of women’s knowledge on MTCT and EMTCT may be due to differences in study periods, locations and sample sizes [16]. The current study found a positive association between marital status and knowledge on MTCT of HIV. Married women and those divorced were more likely to have an all-inclusive knowledge on MTCT and its elimination. This finding is consistent with results from Ethiopia [14, 15] and Nigeria [4, 33]. The plausible explanation could be that married women acquire health information during ANC visits and related family planning services at health care centers [14].

Similarly, women’s access to media (both TV and radio) at least once a week have been found to be correlated with comprehensive MTCT and EMTCT knowledge. Women who listened to radio or watched television at least once a week were more likely to have adequate MTCT knowledge. This finding is consistent with studies from Ethiopia, [14, 34], sub-Saharan Africa [35] and Vietnam [36] where media exposure was associated with increased odds of knowledge on MTCT and its prevention. This may be due to the fact that health education campaigns on HIV and its transmission prevention are carried out through media broadcasting especially radio which has become a very useful medium in disseminating health education information in different local languages to intended targets [37]. However, an inverse association was found between access to mass media and comprehensive MTCT and EMTCT knowledge in studies conducted in Nigeria [4] and Zimbabwe [2].

Furthermore, the region where a woman resided was found to be positively associated to knowledge on MTCT of HIV and its prevention. Women who were living in southern and eastern part of Rwanda have increased odds of comprehensive maternal knowledge on MTCT and EMTCT of HIV compared to those in Kigali. This may be due to inter-regional variations about access to health education and HIV related knowledge as well as resource allocation between the various regions in Rwanda [32, 37]. In addition, women who received post-test counselling had higher odds of having adequate knowledge on MTCT. Similar finding was reported in Zimbabwe [2] where post-test counselling was found to be significantly associated with MTCT knowledge. The reason could be that women who had tested and counselled may be exposed to MTCT information during antenatal and family planning visits. Again, MTCT knowledge have been reported to aid HIV infection awareness among childbearing women thereby enhancing their acceptance and willingness to procure testing, receive test results and post-test counselling [8, 15].

Our study however, found an inverse association between educational level and knowledge on MTCT. Women with higher levels of education in Rwanda were less likely to have adequate knowledge on MTCT and its elimination compared to those with no level of education. This finding is in contrast with those reported in Nigeria [4], Tanzania [8], and Ethiopia [14, 16] where women with primary or higher levels of education have increased odds of having knowledge on MTCT. Also, a study conducted in Zimbabwe found no significant association between childbearing women’s knowledge on MTCT and highest level of education [2]. This apparent contrast may be due to differences in adult literacy rate which is estimated at 73% in Rwanda and 78% in Tanzania [38] and an interplay of geographical variations between these countries. Another plausible reason may be that some financially independent women with higher education may not necessarily access health services at government hospitals but rather at private health facilities where these EMTCT protocols and guidelines may not be fully implemented or followed. Additionally, unlike government or state-owned health facilities, the private medical facilities may not organize health education and promotion sessions or talk for their clientele where information on EMTCT is disseminated. The women even-though highly educated may have limited information and knowledge on EMTCT.

Again, women who lived in rural areas were less likely to have sufficient knowledge on MTCT of HIV compared to those in urban centers in Rwanda. Studies conducted in Tanzania [8], Ethiopia [11, 14, 16], South Africa [39] reported that women who lived in urban areas have increased knowledge on MTCT. The observed disparities (rural/urban) may be due to differences in access to and quality of HIV related information and other resources between these settings. Another plausible reason could be urban dwellers are better exposed to HIV information than rural women who invariably have limited access to EMTCT services. Also, dwelling in rural settings have been found to limit uptake and utilization of EMTCT of HIV services [40, 41]. Interestingly, an inverse association have been found between women who were in the richest quantiles and MTCT knowledge in Rwanda. Women who were from rich households were less likely to have MTCT knowledge compared to those in the poorest quantiles. This finding differs from reports from Tanzania, [8] and sub-Saharan Arica [10] that indicated positive association between women in wealthy households and knowledge on MTCT. The variation may be due to contextual and geographical differences and sample characteristics in our study in comparison to the others. Our study found no association between respondents’ age, employment status, health facility visitation, frequency of reading newspaper amongst others.

### Policy implication of study findings

The health ministry of Rwanda and policy makers should design comprehensive evidence-based education campaigns to enhance women’s knowledge on MTCT of HIV and its elimination. Also, these elaborate and rigorous education campaigns should be carried out using mass media, both radio and television in diverse local languages within the cultural context in order to increase awareness on MTCT and its prevention among women in their reproductive age especially in the rural centers in Rwanda. Health managers should create awareness about HIV self-testing and design programs that incentivizes and enhances self-testing among women in their reproductive age especially for those who could not access traditional testing centers in rural areas. This will help eliminate mother-to-child transmission of HIV. As recommended by the WHO, every woman in their reproductive age must regularly test for HIV. This will help them to know their HIV status so that those who are positive can take the necessary precautions to prevent MTCT, if they decide to become pregnant. Efforts must also be made to help demystify the fears and stigma associated with HIV. EMTCT service providers must educate women on the benefits associated with knowing one’s status.

Furthermore, voluntary counselling and testing services should be made available and easily accessible especially in the rural areas and designed such that it enhances maximum privacy and confidentiality among women in their reproductive age. Adequate logistics and resources such as HIV testing kits, antiretroviral drugs, reagents/buffers, and others should be allocated to service providers especially those in the hinterlands in Rwanda to aid their work and the delivery of EMTCT services. Finally, women who tested positive for HIV should be educated on the need to consider other available modes of safe delivery such as caesarean sections and infant feeding using formulas to help prevent MTCT.

### Strengths and limitations of the study

This current study used a large sample of respondents which ensured statistical power. Also, sample weighting and conducting analysis through complex samples ensured minimal sample biases and produced estimates that were representative of the general population. Furthermore, recall biases associated with DHS surveys may have influenced the study results. The cross-sectional nature limits causation in this study. The use of secondary data in this study may have limited selection and analysis of other critical variables such as distance to voluntary counseling and testing centers, male partners’ knowledge on MTCT and EMTCT which could have impacted the study results. The study findings should be interpreted against these strengths and limitations.

## Conclusion

Findings from this study showed that marital status, frequency of listening to radio and watching television at least once a week, knowledge and use of HIV test kits and receiving post-test counselling were independently associated with comprehensive MTCT and EMTCT knowledge. These factors may be included in the design and implantation of health promotion strategies by policy influencers to enhance women’s knowledge on MTCT and its prevention in Rwanda and contribute to the attainment of universal health coverage and sustainable development goals.

## Data Availability

The dataset analyzed in this study are available in the Demographic and Health Survey (DHS) repository (https://dhsprogram.com/data/available-datasets.cfm).

## Abbreviations

HIV: Human Immunodeficiency Virus
AIDS: Acquired Immune Deficiency Syndrome
MTCT: Mother-to-child Transmission
PMTCT: Prevention of mother-to-child Transmission
RDHS: Rwanda Demographic and Health Survey
LMIC: Lower-and-middle income country
ART: Anti retroviral therapy
WHO: World Health Organization
